# Variation in chemical composition and antimalarial activities of two samples of *Terminalia albida* collected from separate sites in Guinea

**DOI:** 10.1186/s12906-021-03231-3

**Published:** 2021-02-15

**Authors:** Aissata Camara, Mohamed Haddad, Mohamed Sahar Traore, Florence Chapeland-Leclerc, Gwenaël Ruprich-Robert, Isabelle Fourasté, Mamadou Aliou Balde, Jade Royo, Melissa Parny, Philippe Batigne, Marie Salon, Agnès Coste, Aliou Mamadou Balde, Agnès Aubouy

**Affiliations:** 1 UMR 152 PHARMADEV, IRD, UPS, Université de Toulouse, Toulouse, France; 2 Institute for Research and Development of Medicinal and Food Plants of Guinea (IRDPMAG), Dubréka, Guinea; 3 Department of Pharmacy, University Gamal Abdel Nasser of Conakry, Conakry, Guinea; 4 UMR 8236 LIED, CNRS, Université de Paris, Paris, France; 5 Department of Pharmaceutical Sciences, University of Antwerp, Antwerp, Belgium

**Keywords:** *Terminalia albida*, Malaria, Geographical location, UHPLC-HRMS, Plant molecular analysis, *Plasmodium berghei*, *Plasmodium chabaudi*

## Abstract

**Background:**

The disparity of harvesting locations can influence the chemical composition of a plant species, which could affect its quality and bioactivity. *Terminalia albida* is widely used in traditional Guinean medicine whose activity against malaria has been validated in vitro and in murine models. The present work investigated the antimalarial properties and chemical composition of two samples of *T. albida* collected from different locations in Guinea.

**Method:**

*T. albida* samples were collected in different locations in Guinea, in Dubréka prefecture (West maritime Guinea) and in Kankan prefecture (eastern Guinea). The identity of the samples was confirmed by molecular analysis. In vitro antiplasmodial activity of the two extracts was determined against the chloroquine resistant strain PfK1. In vivo, extracts (100 mg/kg) were tested in two experimental murine models, respectively infected with *P. chabaudi chabaudi* and *P. berghei ANKA.* The chemical composition of the two samples was assessed by ultra-high-performance liquid chromatography coupled to high resolution mass spectrometry.

**Results:**

In vitro, the Dubréka sample (*Ta*D) was more active with an IC_50_ of 1.5 μg/mL versus 8.5 μg/mL for the extract from Kankan (*Ta*K). In vivo, the antiparasitic effect of *Ta*D was substantial with 56% of parasite inhibition at Day 10 post-infection in *P. chabaudi* infection and 61% at Day 8 in *P. berghei* model, compared to 14 and 19% inhibition respectively for the treatment with *Ta*K. In addition, treatment with *Ta*D further improved the survival of *P. berghei* infected*-*mice by 50% at Day 20, while the mortality rate of mice treated with *Ta*k was similar to the untreated group. The LC/MS analysis of the two extracts identified 38 compounds, 15 of which were common to both samples while 9 and 14 other compounds were unique to *Ta*D and *Ta*K respectively.

**Conclusion:**

This study highlights the variability in the chemical composition of the species *T. albida* when collected in different geographical locations. These chemical disparities were associated with variable antimalarial effects. From a public health perspective, these results underline the importance of defining chemical fingerprints related to botanical species identification and to biological activity, for the plants most commonly used in traditional medicine.

**Supplementary Information:**

The online version contains supplementary material available at 10.1186/s12906-021-03231-3.

## Background

Malaria, an infectious disease caused by a protozoan of the genus *Plasmodium*, continues to affect many countries in tropical and subtropical Africa [1]. Although significant progress in the fight against malaria has been made, the data reported by the World Health Organization for the period 2015–2017 remain worrying with no reduction in the number of cases worldwide [1]. The resistance developed by the vector to insecticides [2] and the therapeutic failure of antimalarial drugs [3, 4] explain the reasons for the current prevalence of the disease. In addition, access to care for populations living in malaria endemic areas remains a critical problem, especially in sub-Saharan Africa [5].

Consequently, new affordable and effective treatments are needed to reduce malaria in endemic countries. For this purpose, plants are a promising and reliable source. The structural diversity of natural products as well as their ability to interact with therapeutic targets justify their exploration in the search for new drugs. More than 40% of the authorized drugs on the market are of natural or semi-synthetic origin [6]. In addition, the practice of traditional medicine based on the use of plants is largely widespread in local populations in southern countries due to the poor accessibility to primary health care. However, many parameters influence the therapeutic effectiveness of a plant material based on its chemical composition, including environmental and genetic factors, in addition to drying and storage conditions [7]. Because of these disparities, the WHO does not recommend the use of *Artemisia annua* plant material in any form for the treatment or prevention of malaria [8], although the antimalarial activity of this plant has been scientifically validated [9].

Plants of the genus *Terminalia* by their original composition have been reported for their various biological activities, especially in the treatment of malaria [10, 11]. *Terminalia albida* Sc. Elliot (*Combretaceae* family) is widely used in traditional Guinean medicine in the treatment of various diseases including malaria [12]. Its antimalarial properties have been previously demonstrated both in vitro and in vivo. In vitro, two studies have shown that *T. albida* has antiparasitic activities against the chloroquine-resistant strain Pf-K1 with half maximal inhibitory concentrations (IC_50_) of 0.6 μg/mL [13] and 1.5 μg/mL [14]. In the latest study, our team also reported high antimalarial activity in a murine model of experimental cerebral malaria. In this model, untreated mice died by Day 9 post-infection whereas *T. albida* treated-mice were all still alive by Day 12 post-infection. Such activity was associated to anti-inflammatory and anti-oxidant properties of *T. albida* in this study [14].

Here, we postulate that distinct geographical and environmental harvesting locations imply different chemical composition of the same plants. In the present study, our aim was to compare the antimalarial activity of two samples of *T. albida* collected in two distinct geographical and environmental areas of Guinea.

## Methods

### Plant material

*T. albida* wild samples were harvested in May 2018 in two different locations in Guinea in Danaya localized in Dubréka prefecture (sample called *Ta*D) and in Tokounou, a town from the sub-prefecture of Kankan (sample called *Ta*K). Plants were collected in the two botanical gardens of Danaya and Tokonou of the Institute for Research and Development of Medicinal and Food Plants of Guinea (IRDPMAG), one of the research center of the Guinean Ministry of Higher Education and Scientific Research. More than 600 kms separate Danaya from Tokounou. Dubréka area, localized in lower Guinea, is a wetland dominated by mangroves whereas Kankan area is in upper Guinea, a dry area of grassy savannah. A first identification was carried out in the field, then confirmed by the IRDPMAG botany department (Dr Sékou Moussa Keita) where both reference specimens (38HK461 for *Ta*D and 38HK457 for *Ta*K) were deposited. *Terminalia albida* is a plant well known in Guinea because of its wide traditional use especially in Lower, Middle and Upper Guinea. The identification of the plant was based on macroscopic criteria using the basis of morphological characteristics of the plant.

### Molecular analysis

To confirm the identity of the samples, we carried out a molecular comparison of the two plants obtained in Dubreka and Kankan, and morphologically identified as *T. albida*.

As no genomic sequences of *T. albida* are currently available in conventionally used genomic databases, sequences overlapping the 18S ribosomal RNA gene (partial sequence), the internal transcribed spacer 1 (ITS1), the 5.8S ribosomal RNA gene, the internal transcribed spacer 2 (ITS2) and the 26S ribosomal RNA gene (partial sequence) from 6 *Terminalia* species (*T. glaucescens*, GenBank MH432183.1; *T. avicennioides*, GenBank MH432186.1; *T. arenicola*, GenBank MH432184.1; *T. catappa*, GenBank MH432182.1; *T. benzoe,* GenBank MH432178.1 and *T. bellirica*, GenBank KC602394.1) were retrieved from the NCBI website (HTTP://www.ncbi.nlm.nih.gov/). These sequences of 688 to 691 bp were aligned using CLUSTALW [15]. The primer pair TermFor (5′ CCTGCGGAAGGATCATTGTCG 3′) and TermRev (5′ GCTTAAACTCAGCGGGTAGCC 3′) was then designed in strictly identical genomic regions in order to amplify the targeted sequence, including ITS1 and ITS2, in both plants *TaK* and *TaD.* See Additional file 1 for the location of the primers on the targeted ITS region.

Genomic DNA from *TaK and TaD* were extracted with the DNeasy Plant Mini Kit (Qiagen) according to the manufacturer’s instructions. As initial material for DNA extraction, about 100 mg of each sample was ground in liquid nitrogen in a mortar with a pestle to create a fine powder, followed by an additional disruption with the homogenizer Biorad PRECESS24. Amplification of the targeted ITS region in both samples was achieved by PCR using GoTaq polymerase (Promega), by following the recommendations of the supplier and with an optimal annealing temperature of 58 °C. In each case, an amplified fragment of the expected size (about 660 bp) was obtained and then sequenced by a provider (Genewiz). Sequence comparisons between *Terminalia* sp. and the newly sequenced sequences of *Ta*K and *Ta*D have been realized using the CLUSTALW software, as above. Finally, the unique sequence reported in this paper has been deposited in GenBank under the accession number MW443102.

### Preparation of plant extracts

For both samples, the stem barks were dried at room temperature in the laboratory for 14 days and ground into powder. The powdered material (600 g) was macerated with 2 L of pure methanol for 72 h. The macerate was then filtered and dry evaporated under reduced pressure (Büchi® rotary evaporator, model R-200). The yields (Y) were determined by the formula Y = (W2/W1) × 100 where W1 represents the weight of the plant material before extraction and W2 the weight of the dried extract. The dried extracts were stored at − 20 °C until used. For in vivo experiments, 100 mg of crude extracts was dissolved in 27.8 mL of distilled water (3.6 mg/mL of water) before administration by oral gavage.

### Antiplasmodial in vitro activity

In vitro tests were carried out in the Microbiology, Parasitology and Hygiene laboratory of the Department of Pharmaceutical Sciences in Antwerp (Belgium). The activity against the chloroquine and pyrimethamine-resistant *P. falciparum* strain K1 was evaluated using the lactate dehydrogenase procedure previously described by Tuenter et al. [16]. Briefly, *Plasmodium* culture was maintained in RPMI-1640 medium supplemented with 2% penicillin/streptomycin solution, 0.37 mM hypoxanthine, 25 mM HEPES, 25 mM NaHCO_3_, and 10% O^+^ human serum, complemented with 4% human O^+^ erythrocytes. All cultures and assays were conducted at 37 °C under a N_2_-enriched atmosphere (4% CO_2_, 3% O_2_, and 93% N_2_). Stock solutions of extracts (20 mg/ml) were prepared in DMSO (20 mM) and diluted with parasite culture medium. Parasite culture was adjusted to 1% parasitemia and 2% hematocrit and cocultured in presence of increasing dilutions of plant extracts in 96-well tissue culture plates. Test plates were incubated at 37 °C for 72 h before being stored at − 20 °C until further processing. After thawing, 20 μL of hemolyzed parasite suspension from each well was added to 100 μL of Malstat reagent and 10 μL of a 1:1 mixture of phenazine ethosulfate (2 mg/mL) and nitroblue tetrazolium (0.1 mg/mL). The plates were kept in the dark for 2 h, and the change in color was measured spectrophotometrically at 655 nm. The results were expressed as IC_50_ values determined from drug concentration − response curves. Chloroquine diphosphate was used as an antiplasmodial reference drug. Each sample was tested in triplicate.

### Experimental animals and ethical aspects

Healthy male and female C57BL/6 mice aged 7 to 13 weeks and weighing 18 to 22 g were obtained at the PHARMADEV pet store in Toulouse, France. Animal welfare requirements were rigorously followed during the experiments in accordance with the recommendations of the Midi-Pyrénées Ethics Committee for Animal Experiments in Toulouse, France, and by respecting the principles of the 3Rs (**R**eplacement, **R**eduction and **R**efinement). The mice were maintained in collective cages (720 cm^2^, 6 to 10 animals/cage) under standard and constant laboratory conditions (temperature of 23 to 25 °C, 12 h light/darkness cycles 12/12 h and relative humidity around 60%) with free access to food and tap water. The welfare of the animals was enriched with huts and paper towels for making nests. The experiments were carried with a reduced number of animals (6 animals/group). During *Plasmodium* infections and to avoid painful death, mice were monitored daily (weight monitoring, posture, behavior, hair appearance) and euthanized if weight loss was greater than 20% of the initial weight, if the animals showed signs of mutilation, breathing difficulties, or symptoms of paralysis. The study was authorized with the APAFIS permit number # 5921–2,016,070,118,008,477 v3.

### In vivo antimalarial activity of plant extracts

According to the standard 4-day suppressive test [17], the antimalarial effect of the two samples of *T. albida* was measured in murine models. Experimental uncomplicated and cerebral malaria models based respectively on *P. chabaudi chabaudi* AS and *P. berghei ANKA* infections were used (strains were kindly given by A. Berry, CPTP research unit, Toulouse). In C57BL/6 mice, *P. chabaudi chabaudi* strain AS causes non-lethal malaria characterized by a peak of parasitemia around 10 days after infection (Day 10), followed by spontaneous healing [18]. *P. berghei* ANKA induces cerebral malaria which causes 98% mortality in young C57BL/6 mice at 6 to 14 days after infection [19]. Mice were randomly divided into four treatment groups of six mice (3 males and 3 females per group): *Ta*D, *Ta*K, chloroquine and water. Mice were infected intraperitoneally with 200 μL of infected blood containing 10^6^ infected erythrocytes. Two hours after infection, mice were treated by oral gavage for 4 consecutive days (Day 0 to Day 3) with 100 mg/kg of *Ta*D or *Ta*K extract dissolved in distilled water (stock concentrations 3.6 mg/mL), 5 mg/kg of chloroquine dissolved in distilled water (stock concentration 0.2 mg/mL) for the positive control and 25 mL/kg of water for the negative control. According to their weight and the treatment group, mice received 400 to 600 μL of treatment by oral gavage. Mice weight was assessed daily until death or Day 20. The parasitaemia was checked daily until Day 15 by microscopic examination of Giemsa-stained thin blood smears (RAL 555 kit, RAL diagnostics) and calculated as follows: parasitaemia = 100 × (number of parasitized red blood cells / total number of red blood cells counted). The mean percentage of chemosuppression was calculated by the formula [(A - B) / A] × 100, where A is the mean percentage of parasitaemia in the negative control group and B is the mean percentage of parasitaemia in the test group. For *P. berghei* infection, severe symptoms as described above were checked twice a day and mice were euthanized if they presented at least one of the symptoms described above. Survival was determined over a 20-day period and compared between groups. At the end of the study, the mice still alive were euthanized by CO2 inhalation (flow rate of 5 L/min, equal to 36% of the volume of the chamber, maintained for at least 1 min after respiratory arrest).

### UHPLC-HRMS for profiling

UHPLC-HRMS analyses of the methanolic extracts of *T. albida* (1 mg/mL) were performed under the same conditions as those described by Chassagne et al. 2018 [20], on a UHPLC chain consisting of an UltiMate 3000 UHPLC (Thermo Fisher Scientific, United Kingdom) and equipped with a diode-array UV detector (DAD) at wavelengths between 210 and 400 nm. The stationary phase was an Acquity BEH C18 column (100 × 2.1 mm ID, 1.7 μm, Waters, USA). The mobile phase was composed of two solvents: Solvent A: 0.1% formic acid-water; solvent B: 0.1% formic acid-acetonitrile. The following gradient was used (0–0.5 min, 95% A; 0.5–12 min, 95–5% A; 12–15 min, 5% A; 15–15.5 min, 5–95% A; 15.5–19 min, 95% A). The injected volume was 2 μl and the column temperature was maintained at 40 °C. The flow rate was set at 0.3 ml/min. The mass spectra were performed on a UHPLC-DAD-LTQ Orbitrap XL mass spectrometer (Thermo Fisher Scientific, UK) equipped with an electrospray ionization system (ESI) in negative (NI) and positive (PI) mode and recorded between 100 and 1500 Da. The main peaks are described in *m/z* ratio.

### Peak analysis

MS-DIAL software 3.50 was used to process the raw UHPLC/HR-MS data. In positive ionization mode, the mass spectra were extracted between 100 and 1500 Da following the automatic detection of the indices performed between 0.3 and 17.0 min. Tolerances were set at 0.01 and 0.40 Da for MS1 and MS2 respectively in centroid mode. The data of the generated spectra were exported to Microsoft Excel with the exception of peaks detected in the blank sample. In positive ion mode, 2036 peaks were detected for TaK and 1984 peaks for TaD. In negative ion mode, 2434 peaks were detected for TaK and 2400 for TaD. Following the method described by Piskounova et al. [21], the differential analysis was performed by adding to all measurements half of the smallest non-zero value for data containing the value “zero” before the log transformation. Data were exported in comma-separated value (CSV) format for multi-group analysis prior to analysis using MetaboAnalyst [22].

### Significant features identification

Following the method described previously [23], MS-FINDER-RIKEN PRIMe version 3.12 was used to calculate the molecular formula as well as the significant structural features. Bond dissociation energies, mass accuracy, bonds between fragments and nine hydrogen rearrangement rules were taken into account to assign a score to each compound. To limit the number of potential candidates, we used different parameters including: elements comprising exclusively C, H, O; mass tolerance set at 10 ppm and isotope ratio tolerance set at 20%. The SMILES entry in the Natural Products Dictionary (CRC Press v 26:2) and the integrated MS-FINDER system: Universal Natural Products Database (UNPD), KNApSAcK, CheBI (Chemical Entities of Biological Interest), NANPDB (Northern African Natural Products Database), and PlantCyc were used to restrict the search to plant compounds in the database containing their source following the SciFinder search. Analyses were only performed on a list of compounds with a score greater than 7.

### Statistical analysis

The results were analyzed using Graph Pad Prism version 6 software. For the experimental approach, parasitemia and survival were the primary outcomes assessed. Difference in bodyweight was a secondary outcome. The comparisons were made using a unidirectional analysis of variance (ANOVA) followed by Bonferroni’s mean multiple comparison method. The differences were considered significant if *P* < 0.05.

## Results

### Molecular comparison of TaD and TaK

PCR amplifications with primer pair TermFor/TermRev of genomic DNA from *Ta*K and *Ta*D allowed us to obtain a 667 bp and 668 bp PCR fragment, respectively. Sequence alignments are shown in Additional file 1. Alignment of both sequences with each other showed that *Ta*K and *Ta*D sequences are unambiguously identical with 100% identity. So, based upon morphological analysis we can conclude here that *Ta*K and *Ta*D could be both identified as *T. albida*. However, alignment of both sequences with those of 6 other species of *Terminalia*, showed that sequence of *T. albida* can be distinguished from other *Terminalia* sp. with only some slight nucleotide polymorphisms. *T. albida* ITS sequence shows a very high percentage of identity with those of *T. avicennioides* and *T. glaucescens* (99.85%), with only one different nucleotide over the 668 bp. This percentage is slightly lower when compared to ITS sequence from *T. benzoe*, *T. catappa*, *T. arenicola* and *T. bellirica* (96.26, 95.67, 95.37 and 91.03% respectively).

### In vitro and in vivo antiplasmodial activity of *Ta*D and *Ta*K extracts

**Fig. 1:**
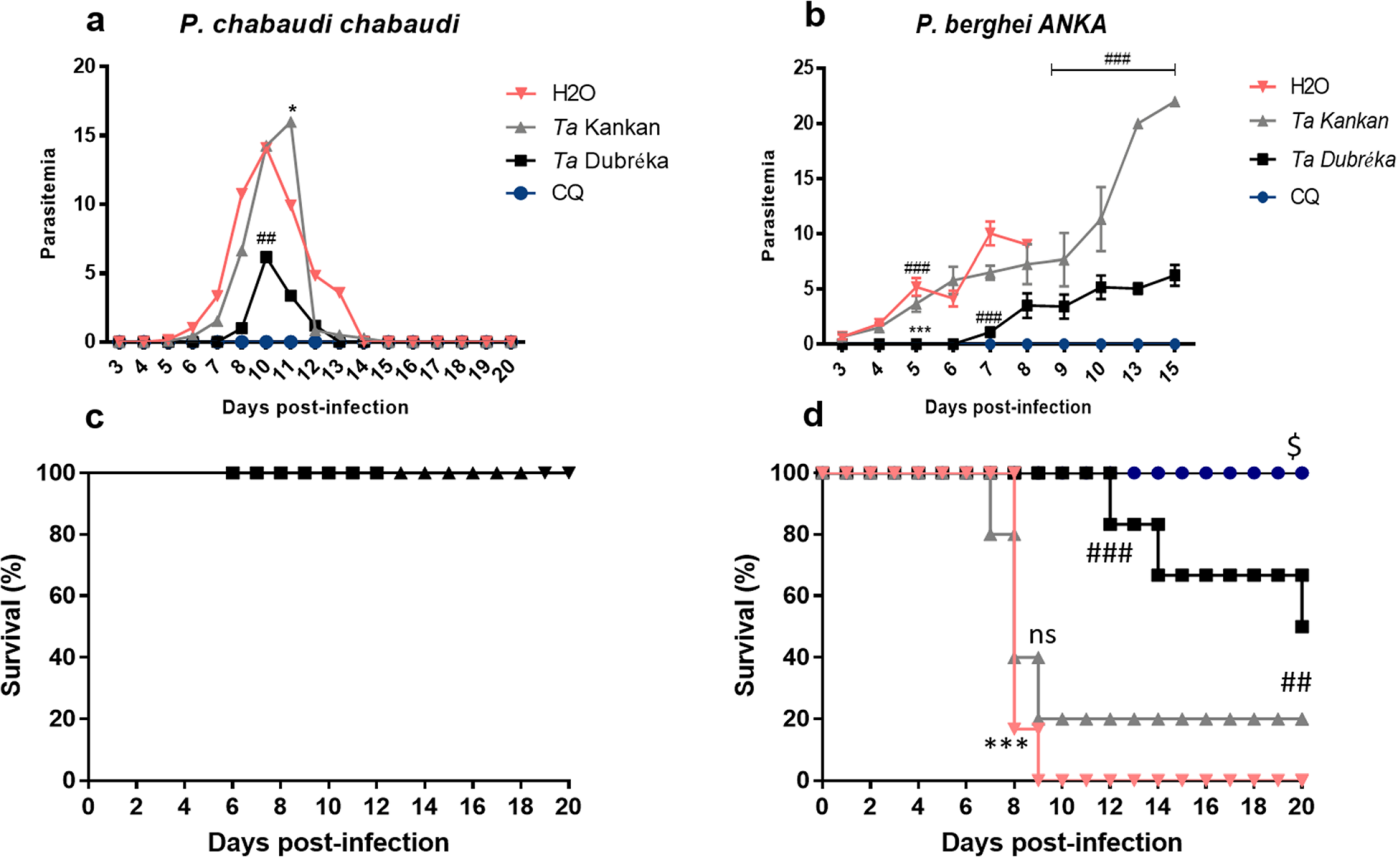
Effect of methanolic extracts of *Terminalia albida* from Kankan (*Ta*K) or Dubréka (*Ta*D) in *Plasmodium berghei ANKA* and *P. chabaudi chabaudi* infection. According to the 4-Day suppression test, C57BL/6 mice (*n* = 6 per group) were infected with 200 μl of parasitic suspension (10^6^/mouse) at Day 0 and treated 2 h later with 100 mg/kg of stem bark of *T. albida* (*Ta*D or *Ta*K), or 5 mg/kg chloroquine, or 25 mL/kg of water from Day 0 to Day 3. **a**, **b** Parasitemia over time. **c**, **d** Survival over time. The comparisons were made using a unidirectional analysis of variance (ANOVA) followed by Bonferroni’s mean multiple comparison method. ^#^ Comparison between *Ta*D and *Ta*K, * comparison to H_2_O, ^$^ comparison to CQ. One symbol means *P* < 0.05, two symbols mean *P* < 0.005, three symbols mean *P* < 0.0005, ns means not significant

Extraction yields from our *Terminalia* root bark samples were 47.6% for Dubréka and 53.3% for Kankan. The extracts were evaluated in vitro against the chloroquine resistant strain PfK1 to measure their ability to inhibit parasitic growth. *Ta*D showed a remarkable inhibitory activity against PfK1 with an IC_50_ of 1.50 μg/mL while the mean inhibitory concentration of *Ta*K was 8.52 μg/mL (Table 1). To compare in vivo activity of both *T. albida* samples, mice infected with *P. chabaudi* and *P. berghei* were treated with *Ta*D or *Ta*K extract at a dose of 100 mg/kg. The positive control group received chloroquine and the negative control group received water. In the *P. chabaudi* model, treatment with *Ta*D resulted in a significant reduction of 56% parasitemia at Day 10, corresponding to the peak of parasitemia in the negative control group (Table 1 and Fig. 1a). Conversely, treatment with *Ta*K decreased *P. chabaudi* parasitaemia of 14% only at Day 10, as shown on Table 1 and Fig. 1a. In the *P. berghei* model, *Ta*D treatment resulted in 100% inhibition of the parasitemia at Day 5, 89% at Day 7 and 61% at Day 8. With *Ta*K, parasitemia was reduced by 63% at Day 5, 35% at Day 7 and 19% only at Day 8 (Table 1, Fig. 1b).

**Table 1:** In vitro and in vivo antiplasmodial activity of crude extract of *Terminalia albida* stem bark from Dubréka (*Ta*D) and Kankan (*Ta*K), Guinea

	In vitro IC_50_ (μg/mL)	% Parasite suppression (± SD)			
	against *P. falciparum* K1	against *P. chabaudi*	*against P. berghei*		
		Day 10	Day 5	Day 7	Day 8
CQ	0.09 ± 0.5	100	100	100	100
*Ta*D	1.5 ± 0.4	56 ± 0.6	100	89 ± 0.3	61 ± 1.0
*Ta*K	8.5 ± 1.2	14 ± 1.4	63 ± 0.7	35 ± 0.4	19 ± 1.27

*CQ* chloroquine (5 mg/kg), *TaD* and *TaK* (100 mg/kg), *SD* standard deviation

### Differences in mice survival and bodyweight during infection after *Ta*D and *Ta*K treatment

**Fig. 2:**
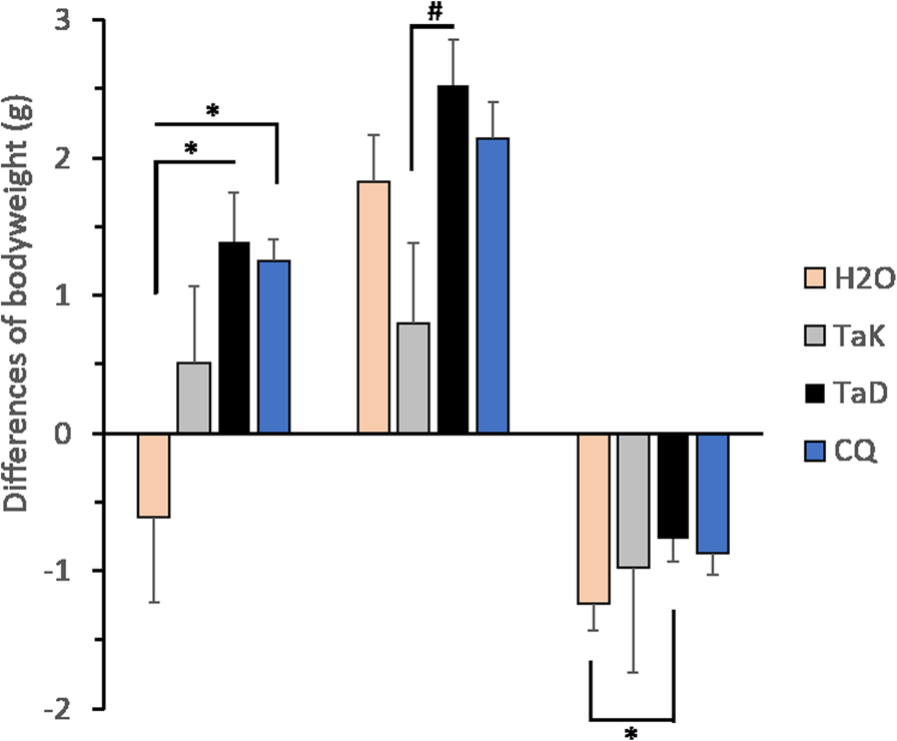
Differences of bodyweight of *Plasmodium* infected-C57BL/6 mice before (Day 0) and after (Day 7, Day 12 or Day 20) treatment with methanolic extracts of *Terminalia albida* stem bark from Kankan (*Ta*K) or Dubréka (*Ta*D). Bodyweight was measured in grams. Mice (*n* = 6 per group) were infected and treated as detailed in Fig. 1. Pcc means *P. chabaudi chabaudi*, PbA means *P. berghei* ANKA. The comparisons were made using a unidirectional analysis of variance (ANOVA) followed by Bonferroni’s mean multiple comparison method. ^#^ Comparison between *Ta*D and *Ta*K, * comparison to H_2_O. One symbol means P < 0.05

No death was recorded after *P. chabaudi* infection whatever the group of treatment (Fig. 1c). This result confirms the absence of toxicity of both extracts and indicates the safety of the administered dose. Conversely, in *P. berghei* infected-mice, *Ta*K did not improve mice survival (Fig. 1d). At Day 8, death was recorded in 4/6 mice that received *Ta*K treatment and in 5/6 untreated mice while no death occurred in the *Ta*D group, nor in the CQ group (*P* < 0.0005 for *Ta*D versus H_2_O or *Ta*K). At Day 9, 5/6 mice that received *Ta*K were dead. In the *Ta*D group, deaths occurred from D12 but by Day 20, the survival rate was still 50% (Fig. 1d). Weight loss was also compared between treatments groups during infection. As shown in the Fig. 2, all *P. chabaudi* infected-mice gained weight except the untreated group at Day 12. *Ta*D and CQ treatments led to higher bodyweight gain resulting in significant differences with the water group (*P* < 0.05 for *Ta*D and CQ versus H_2_O). In addition, at Day 20, the mean bodyweight of the *Ta*D treated-mice was higher than the *Ta*K treated-mice (P < 0.05 *Ta*D versus *Ta*K). In the *P. berghei* model, *Ta*D treatment prevented excessive weight loss compared to the untreated batch with a significant difference observed (*P* = 0.03 for *Ta*D versus H_2_O).

### Metabolite profiling of the two *T. albida* samples *Ta*D and *Ta*K

**Fig. 3:**
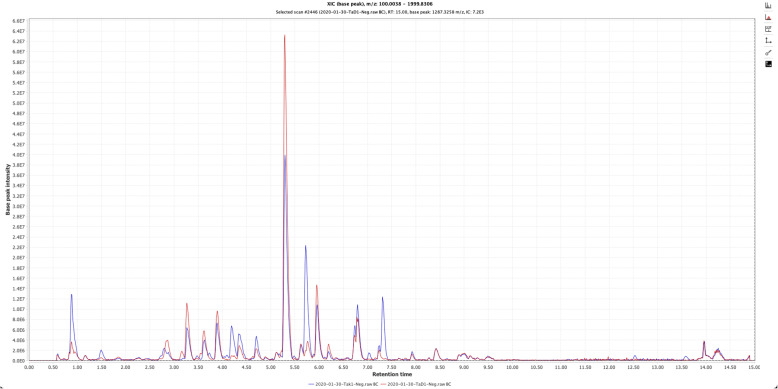
Base peak chromatograms in negative ion mode obtained for the root extract of *T. albida* collected in Danaya, Préfecture of Dubréka (red) and in Tokounou, sub-prefecture of Kankan (blue)

**Fig. 4:**
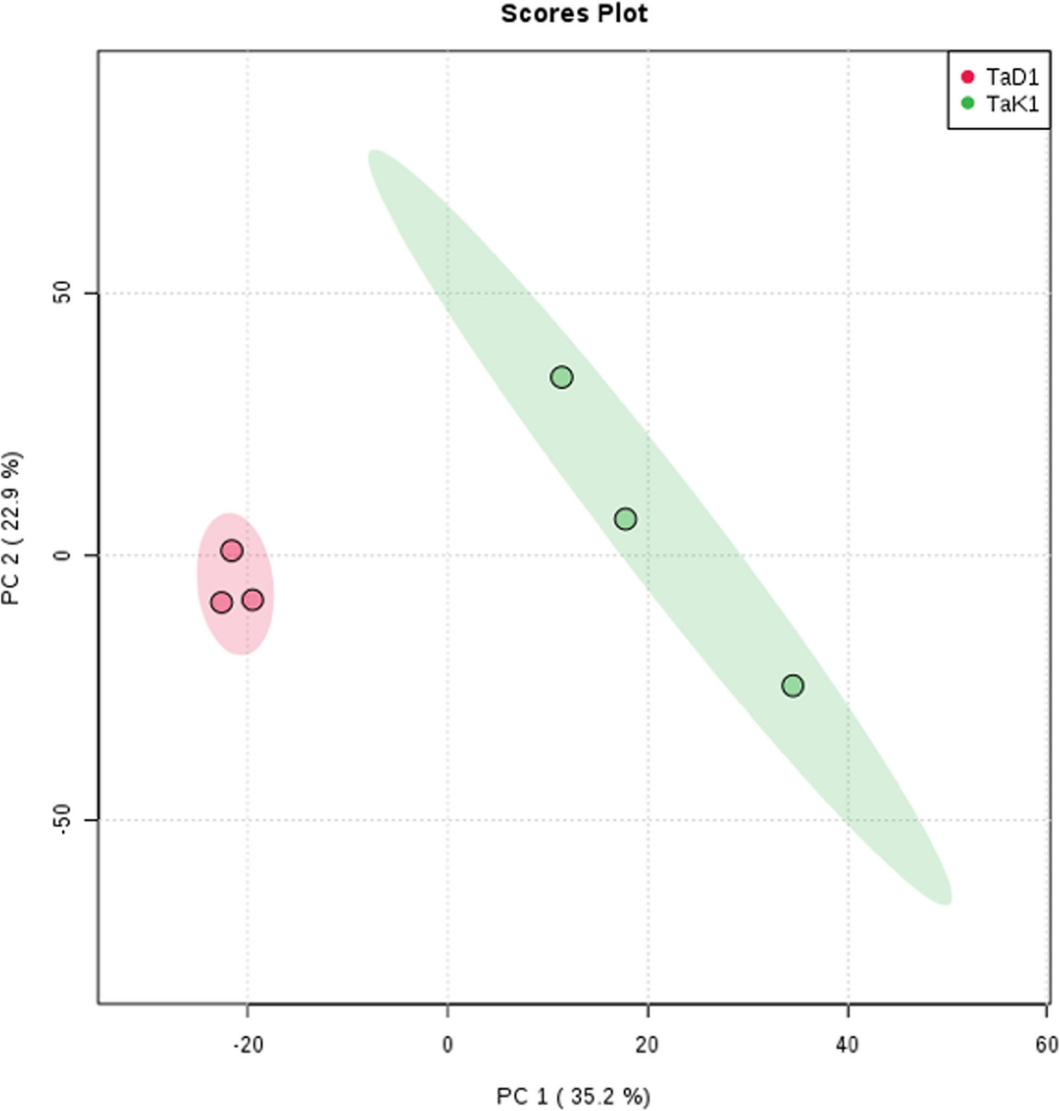
LC-MS PCA analysis (unsupervised multivariate analysis approach) of extracts from TaK and TaD (regions display of 95% confidence), performed using MetaboAnalyst, to determine differences between samples, based on their metabolite profiles, considering each location separately

Metabolite profiling of *Ta*D and *Ta*K by LC-HRMS was acquired in positive and negative ionization mode (Fig. 3). From these two samples, the qualitative analysis by LC-HRMS allowed to identify putatively 38 compounds found in either or both samples, through HRMS and MS/MS fragmentation patterns using MS-finder and DNP database. Nine of the 38 annotated compounds were found only in *Ta*D, 14 only in *Ta*K and 15 in both samples. These results are summarized in the Table 2. The MS-Finder dereplication method allowed to annotate these major peaks, mostly found in the *Combretaceae* family. An unsupervised multivariate analysis approach, principal component analysis (PCA), was performed using MetaboAnalyst [21] to determine differences between samples, based on their metabolite profiles, considering each location separately. The score plots are shown in Fig. 4. For the LC-MS data, 58.1% of the variance is explained by the first two principal components. Fine clustering can be observed for the extracts from the two locations, demonstrating a clear separation between the two geographical origins (Fig. 4). This separation of clusters suggests phytochemical differences between plants of each region.

**Table 2:** Putative identified features (m/z × RT pairs) using HRMS and MS/MS fragmentation patterns using MzMine, MS-finder and DNP database

ID	RT (min)	m/z	Δ Da	Formula finder	Putative ID	Ontology	Detected in	
							*Ta*D	*Ta*K
1	2.69	469.0038 [M-H] -	−0.0003489	C_21_H_10_O_13_	flavogallonic acid	Hydrolyzable tannins	•	•
2	5.9	641.3692 [M + H] +	0.0014128	C_37_H_52_O_9_	23-Galloylarjunolic acid	Triterpenoids	•	•
3	4.95	315.0139 [M-H] -	−0.0018512	C_15_H_8_O_8_	3-O-Methylellagic acid	Hydrolyzable tannins	•	•
4	6.61	345.061 [M + H] +	0.0016802	C_17_H_12_O_8_	Nasutin B	Hydrolyzable tannins	•	•
5	3.36	633.0721 [M-H] -	−0.0004476	C_27_H_22_O_18_	Corilagin	Hydrolyzable tannins	•	•
6	6.01	519.3322 [M + H] +	0.0014948	C_30_H_46_O_7_	Cucurbitacin F	Cucurbitacins	•	•
7	4.27	300.9984 [M-H] -	0.0007995	C_14_H_6_O_8_	Ellagic acid	Hydrolyzable tannins	•	•
8	4.11	447.0561 [M-H] -	0.001142	C_20_H_16_O_12_	Eschweilenol C	Hydrolyzable tannins	•	•
9	5.85	487.3421 [M + H] +	0.0019794	C_30_H_46_O_5_	Astrantiagenin J	Triterpenoids	•	•
10	7.04	547.3265 [M-H] -	0.0009789	C_31_H_48_O_8_	Quadrangularic acid F	Cycloartanols and derivatives	•	•
11	5.3	817.4001 [M-H] -	− 0.000099	C_43_H_62_O_15_	Quadranoside XI	Triterpene saponins	•	•
12	5.65	521.0927 [M-H] -	− 0.0000521	C_23_H_22_O_14_	Quercetagetin 7-(6″-acetylglucoside)	Flavonoid-7-O-glycosides	•	•
13	5.61	667.4061 [M + H] +	− 0.0015523	C_36_H_58_O_11_	Quercilicoside A	Triterpene saponins	•	•
14	5.9	801.405 [M-H] -	0.0008995	C_43_H_62_O_14_	Russelioside G	Steroidal glycosides	•	•
15	3.97	600.9883 [M-H] -	−0.0002343	C_28_H_10_O_16_	Terminalin	Hydrolyzable tannins	•	•
16	12.17	754.585 [M + H] +	0.0016545	C_43_H_79_NO_9_	Chrysogeside D	Fatty acyl glycosides of mono and disaccharides	•	°
17	1.15	214.09 [M + H] +	−0.0009109	C_13_H_11_NO_2_	Bruceolline E	Indoles	•	°
18	5.19	505.3526 [M + H] +	−0.0010109	C_30_H_48_O_6_	Myrianthic acid	Triterpenoids	•	°
19	7.67	489.3578 [M + H] +	0.0012948	C_30_H_48_O_5_	Arjunolic acid	Triterpenoids	•	°
20	4.61	435.1287 [M-H] -	0.0003775	C_21_H_24_O_10_	Phlorizin	Flavonoid O-glycosides	•	°
21	3.46	759.1191 [M-H] -	−0.000299	C_37_H_28_O_18_	Prodelphinidin A2 3′-gallate	Biflavonoids and polyflavonoids	•	°
22	7.8	529.3167 [M-H] -	0.0013078	C_31_H_46_O_7_	Acinospesigenin B	Triterpenoids	•	°
23	3.8	457.0765 [M-H] -	0.0012078	C_22_H_18_O_11_	Epigallocatechin gallate	Catechin gallates	•	°
24	0.92	783.0694 [M + H] +	0.0006408	C_34_H_22_O_22_	Punicalin	Hydrolyzable tannins	•	°
25	7.19	505.3526 [M + H] +	0.0009774	C_30_H_48_O_6_	Protobassic acid	Triterpenoids	°	•
26	7.48	359.0767 [M + H] +	0.0012374	C_18_H_14_O_8_	5,3′-Dihydroxy-4′,5′-dimethoxy6,7methylenedioxyisoflavone	3′-hydroxy, 4′-methoxyisoflavonoids	°	•
27	5.56	329.0296 [M + H] -	−0.003745	C_16_H_10_O_8_	3,3′-di-O-Methylellagicacid;3,3′-Dimethoxyellagic acid	Hydrolyzable tannins	°	•
28	2.2	933.0623 [M + H] -	0.0003774	C_41_H_26_O_26_	Vescalagin	Hydrolyzable tannins	°	•
29	3.79	1083.0574 [M + H] -	0.0011788	C_48_H_28_O_30_	Punicalagin	Hydrolyzable tannins	°	•
30	4.57	450.9933 [M + H] -	−0.0004062	C_21_H_8_O_12_	Flavogallol	Hydrolyzable tannins	°	•
31	6.46	327.014 [M + H] -	0.0006908	C_16_H_8_O_8_	Pteleoellagic acid		°	•
32	7.67	533.348 [M + H] -	0.0007408	C_31_H_50_O_7_	Methyl 4,23, 29-trihydroxy-3,4-seco-olean-12-en-3-oate-28-oic acid	17-hydroxysteroids	°	•
33	3.67	463.0507 [M + H] -	−0.0007903	C_20_H_16_O_13_	Ellagic acid glucoside	Hydrolyzable tannins	°	•
34	4	433.0403 [M + H] -	0.0011876	C_19_H_14_O_12_	Ellagic acid arabinoside	Hydrolyzable tannins	°	•
35	6.26	817.4003 [M + H] -	0.0027568	C_43_H_62_O_15_	Quadranoside XI;(+) - Quadranoside XI	Triterpene saponins	°	•
36	6.24	627.0978 [M + H] -	0.0010289	C_29_H_24_O_16_	1-O-p-(E)-Coumaroyl-4,6-(S)-HHDP-beta-D-glucopyranose	Hydrolyzable tannins	°	•
37	7.2	503.3364 [M + H] -	0.0010639	C_30_H_48_O_6_	Tomentosic acid	Triterpenoids	°	•
38	3.19	483.077 [M + H] -	0.0016802	C_20_H_20_O_14_	1,6-Digalloyl-beta-Dglucopyranose	Tannins	°	•

• Product present

° Product absent

## Discussion

Although the plant kingdom is a promising source for new active substances, the variability of chemical composition in the same species of different origin can affect its quality and bioactivity [24]. Few studies have addressed this problem or reported differences in composition and/or activity between the same species from different areas. Here, we evaluated the antiplasmodial activity of *T. albida* harvested in Guinea in two different areas, Dubréka and Kankan, characterized by different geographical facies (wetland with mangroves versus grassy savannah). To the best of our knowledge, this is the first report on the comparison of plants of the genus *Terminalia* belonging to the same species but from two distinct geographical areas. However, this work is based on the comparison of a single specimen from each area. A study based on the comparison of several specimens from each zone is necessary to strengthen the robustness of these results.

Only 6 species of *Terminalia* are currently available in the genomic database from NCBI. It concerns a common sequence of about 690 bp overlapping in particular ITS1 and ITS2 that are commonly used to compare close species in phylogenetic analysis. Such a sequence allowed us to clearly show that *Ta*K and *Ta*D belong to the same species (meaning *T. albida*, based upon morphological analysis) that is closely related to other *Terminalia* species previously analyzed. Further investigations will be necessary to better understand the phylogenetic organization between *Terminalia* species. Moreover, such approaches could be helpful in the deployment of recent biological tools, as plant DNA barcodes (based upon various markers as ITS2), which are promising, especially for taxonomic discovery [25].

Regarding the antiplasmodial and antimalarial activity, the Dubréka sample (*Ta*D) was much more active than the Kankan sample (*Ta*K). In vitro, *Ta*D displayed an IC_50_ value of 1.5 μg/ml against the PfK1 strain with, while *Ta*K showed only a promising activity with an IC_50_ of 8.5 μg/ml. Similarly, in vivo, *Ta*D treatment showed a promising therapeutic effect in both uncomplicated and cerebral murine malaria models, whereas *Ta*K was ineffective. In *P. chabaudi* infected-mice, parasitemia was inhibited by 56% after *Ta*D treatment, whereas *Ta*K treated-mice presented similar parasitemia to untreated mice. Regarding the experimental cerebral malaria model, *Ta*K also showed low antiplasmodial activity compared to *Ta*D which limited parasite growth from 89% at Day 7 compared to 35% for *Ta*K. In addition, treatment with *Ta*D prevented premature death in mice and maintained 50% survival up to Day 20 unlike *Ta*K whose treatment had no effect on death prevention in this model. Such level of antimalarial activity is noteworthy compared to that of others reported for plants of the genus *Terminalia* in previous studies [10, 11, 26]. Only the stem bark of *T. avicennioides* showed a significant inhibition of 82% of *P. berghei* parasitemia at Day 5 post-infection at 100 mg/kg [11]. As we have previously shown, the capacity of *Ta*D to limit death in *P. berghei*-infected mice may be due to its anti-inflammatory and antioxidant properties [14]. In this model, mice death is attributed to preferential sequestration of leukocytes in the brain, vascular obstruction, endothelial activation and neuroinflammation [19, 27]. The divergence of antimalarial and antiplasmodial activity between our two samples *Ta*D and *Ta*K could be due to the disparity in harvesting locations. Dubréka is located in a wetland area dominated by mangroves that consists of well-irrigated land, while Kankan, in the vast plateau area, is less irrigated and therefore less favorable to optimal plant growth [12]. Interestingly, the use of *T. albida* as monotherapy to treat malaria is most common in lower and middle Guinea than in upper Guinea where Kankan is found (IRDPMAG Report).

To go further, we conducted a preliminary chemical comparative analysis of the samples to find more elements explaining the different antimalarial activities. The qualitative analysis by LC-HRMS of the two extracts reveals many differences in regards of their chemical contents (Table 2, Figs. 3 and 4). This can be explained by the fact that during normal plant development, growth is not constant and chemical composition can be expected to vary. Metabolite composition is strongly affected by genotype, environment, and interactions between genotype and environment, although the extent of variation caused by these factors may depend upon the type of metabolite. Plants produce a large number of metabolites with various structures that play important roles in plant growth, development and response to environments. The plant kingdom may contain between 90,000 and 200,000 metabolites although for a single species, this number can reach several thousand. Ecological conditions and developmental senescence have a significant influence on the physiological metabolism of plants, resulting in a series of adjustments in their metabolic and physiological functions to adapt to environmental changes. Therefore, environmental factors may influence types and contents of active substances, that can explain the chemical differences between *Ta*D and *Ta*K. It was reported by Liu et al. in 2016 that altitude, temperature, sunshine duration and average annual precipitation are important environmental factors that positively or negatively influence the active substance content (tannin, total flavonoids, rutin and total phenolics) and antioxidant activity of *Potentilla fruticosa* L. harvested in different regions of China [28]. Recently, some authors have demonstrated the relationship between efficacy, chemical constituents and distribution of *Artemisia annua* L. collected from different geographical regions in China [29].

Nevertheless, relatively little is known about the influence of geographical distribution on dynamic changes in plant. Despite qualitative LC-HRMS’ analysis of the two extracts *Ta*D and *Ta*K, it seems very difficult to determine which molecule exerts biological activity, since some unknown molecules have not been annotated or even detected by our method. In addition, biological activity can also be linked to the combined effect of several molecules through synergy. In order to go further in this analysis, it would be interesting to apply the methodology described by Chassagne et al. in order to direct hypotheses towards a class of molecule or even to attribute the activity to a given molecule [20]. In addition, other studies have already shown significant variability in phytochemical compounds and bioactivity of identical species of different origin [7, 24].

## Conclusion

This study attempted to highlight the influence of the harvest site on the chemical composition and bioactivity of a plant species. Our data show that the production of secondary metabolites by *T. albida* is highly dependent on its environment. For this bioactive plant, such chemical variability was associated to various bioactivity. Such variability must be taken into consideration to ensure the quality and efficacy of herbal medicines. Chemical patterns defined by UHPLC-HRMS analysis may be used to define bioactive plants.

## Supplementary Information

**Additional file 1:.**

## Data Availability

The datasets used and/or analysed during the current study are available from the corresponding author on reasonable request.

## Abbreviations

*Ta*D: *T. albida* from Dubréka
*Ta*K: *T. albida* from Kankan
IC_50_: 50% inhibitory concentration
LC/MS: liquid chromatography-mass spectrometry
