# Supplementary material for: Variation in chemical composition and antimalarial activities of two samples of Terminalia albida collected from separate sites in Guinea
Source: BMC Complement Med Ther. 2021 Feb 15;21:64. doi: 10.1186/s12906-021-03231-3 (PMC7885413; doi:10.1186/s12906-021-03231-3)
Supplement: Supplementary file 1 — Additional file 1:. [file 12906_2021_3231_MOESM1_ESM.docx]

**Additional file 1**

Alignment using CLUSTALW of sequences overlapping the 18S ribosomal RNA gene (partial sequence), the internal transcribed spacer 1 (ITS1), the 5.8S ribosomal RNA gene, the internal transcribed spacer 2 (ITS2) and the 26S ribosomal RNA gene (partial sequence) from *Ta*K and *Ta*D, as well as from 6 *Terminalia* species retrieved from the NCBI website. Location of primer pair TermFor/TermRev is indicated.

TermFor

10 20 30 40 50 60

| | | | | |

*Ta*D ---CCTGCGGAAGGATCATTGTCGACACCTGCAAAGCAGAGCGACCCGCGAACCGTCTTT

*Ta*K ---CCTGCGGAAGGATCATTGTCGACACCTGCAAAGCAGAGCGACCCGCGAACCGTCTTT

*T. glaucescens* GAACCTGCGGAAGGATCATTGTCGACACCTGCAAAGCAGAGCGACCCGCGAACCGTCTTT

*T. avicennioides* GAACCTGCGGAAGGATCATTGTCGACACCTGCAAAGCAGAGCGACCCGCGAACCGTCTTT

*T. arenicola* GAACCTGCGGAAGGATCATTGTCGACACCTGCAAAGCAGAGCGACCCGCGAACCGTTTTC

*T. catappa* GAACCTGCGGAAGGATCATTGTCGACACCTGCAAAGCAGAGCGACCCGCGAACCGTTTTT

*T. benzoe* GAACCTGCGGAAGGATCATTGTCGACACCTGCAAAGCAGAGCGACCCGCGAACCGTTTTT

*T. bellirica* GAACCTGCGGAAGGATCATTGTCGATACCTGCAGAGCAGAACGACCCGCGAACCGTTTTC

********************** ******* ****** *************** **

Prim.cons. GAACCTGCGGAAGGATCATTGTCGACACCTGCAAAGCAGAGCGACCCGCGAACCGT2TTT

70 80 90 100 110 120

| | | | | |

*Ta*D CAA-TTCCCGGGACACCGGGGGGCGCCCAGCCGCCCGGTAGCCCGAAAGCTCCGGACGCT

*Ta*K CAA-TTCCCGGGACACCGGGGGGCGCCCAGCCGCCCGGTAGCCCGAAAGCTCCGGACGCT

*T. glaucescens* CAA-TTCCCGGGACACCGGGGGGCGCCCAGCCGCCCGGTAGCCCGAAAGCTCCGGACGCT

*T. avicennioides* CAA-TTCCCGGGACACCGGGGGGCGCCCAGCCGCCCGGTAGCCCGAAAGCTCCGGACGCT

*T. arenicola* CAA-TGCCCGGGATACCAGGGGGTGCCTATCCGCCCGGTAGCCCGAAAGCTCCGGACGCT

*T. catappa* TAAATGCCCGGGATACCGGGGGGCGCCTATCTGCCCGGTAGCCCGAAAGCTCCGGACGCT

*T. benzoe* TAAATGCCCGGGATACCGGGGGGCGCCTAGCCGCTCGGTAGCCCGAAAGCTCCGGACGCT

*T. bellirica* CAA-CACCCGGGACACCGGGGGGCGTCCAGCCGCTCGGTAGCCC-AAGGCTCCGGACGCC

** ******* *** ***** * * * * ** ********* ** ***********

Prim.cons. CAAATTCCCGGGACACCGGGGGGCGCCCAGCCGCCCGGTAGCCCGAAAGCTCCGGACGCT

130 140 150 160 170 180

| | | | | |

*Ta*D GGGGGTGCAACCCACCCCC-AGCGAACGGATCTCCGAACAAACCCCGGCGCGCGAAGCGC

*Ta*K GGGGGTGCAACCCACCCCC-AGCGAACGGATCTCCGAACAAACCCCGGCGCGCGAAGCGC

*T. glaucescens* GGGGGTGCAACCCACCCCC-AGCGAACGGATCTCCGAACAAACCCCGGCGCGCGAAGCGC

*T. avicennioides* GGGGGTGCAACCCACCCCC-AGCGAACGGATCTCCGAACAAACCCCGGCGCGCGAAGCGC

*T. arenicola* AGGGGTGCAACCCACCCCCCAGCGGACGAAGCTCCAAACAAACCCCGGCGTGCAAAGCGC

*T. catappa* AGGGGTGCAACCCACCCCCCAGCGGACGAAGCTCCAAACAAACCCCGGCGCGCGAAGCGC

*T. benzoe* AGGGGTGCAACCCACCCTC-AGCGGACGGAGCTCCAAACAAACCCCGGCGCGCGAAGCGC

*T. bellirica* GAGGGTGCAACCCACCTCC-GGCGGATGGAGCTTCAAACAAACCCCGGCGCGCAAAGCGC

************** * *** * * * ** * ************** ** ******

Prim.cons. GGGGGTGCAACCCACCCCCCAGCG2ACGGA2CTCC2AACAAACCCCGGCGCGCGAAGCGC

190 200 210 220 230 240

| | | | | |

*Ta*D CAAGGTACTCTAACGATACGGCATGCGCCCGTAGCCCTGGGTTCCAGTGTGCTCGGGCTG

*Ta*K CAAGGTACTCTAACGATACGGCATGCGCCCGTAGCCCTGGGTTCCAGTGTGCTCGGGCTG

*T. glaucescens* CAAGGTACTCTAACGATACGGCATGCGCCCGTAGCCCTGGGTTCCAGTGTGCTCGGGCTG

*T. avicennioides* CAAGGTACTCTAACGATACGGCATGCGCCCGTAGCCCTGGGTTCCAGTGTGCTCGGGCTG

*T. arenicola* CAAGGTACTCCAACTGTAGGGCATGCGCCCGTAGCCCTGGGTTCCAGTGTGCTCGGGCTG

*T. catappa* CAAGGTACTCCAACGGTAGGGCATGCGCCCGTAGCCCTGGGTTCCAGTGTGCTCGGGCTG

T*. benzoe* CAAGGTACTCCAACGATAGGGCATGCGCCCGTAGCCCTGGGTTCCAGTGTGCTCGGGCTG

*T. bellirica* CAAGGTACTCCAACAAAAGGGCATGCGCCCGTAGCCCTGGGTTCCAGTGCGCTCGGGCTG

********** *** * ****************************** **********

Prim.cons. CAAGGTACTC2AACGATA2GGCATGCGCCCGTAGCCCTGGGTTCCAGTGTGCTCGGGCTG

250 260 270 280 290 300

| | | | | |

*Ta*D CTGTTCGACATCATAAAGTCTAAACGACTCTCGGCAACGGATATCTCGGCTCTCGCATCG

*Ta*K CTGTTCGACATCATAAAGTCTAAACGACTCTCGGCAACGGATATCTCGGCTCTCGCATCG

*T. glaucescens* CTGTTCGACATCATAAAGTCTAAACGACTCTCGGCAACGGATATCTCGGCTCTCGCATCG

T. avicennioides CTGTTCGACATCATAAAGTCTAAACGACTCTCGGCAACGGATATCTCGGCTCTCGCATCG

*T. arenicola* CTGTTCAACATCATAAAGTCTAAACGACTCTCGGCAACGGATATCTCGGCTCTCGCATCG

*T. catappa* CTGTTCAACATCATAAAGTCTAAACGACTCTCGGCAACGGATATCTCGGCTCTCGCATCG

*T. benzoe* CTGTTCAACATCATAAAGTCTAAACGACTCTCGGCAACGGATATCTCGGCTCTCGCATCG

*T. bellirica* CTGTTCGATGCGATAAAGTCTAAACGACTCTCGGCAACGGATATCTCGGCTCTCGCATCG

****** * ************************************************

Prim.cons. CTGTTCGACATCATAAAGTCTAAACGACTCTCGGCAACGGATATCTCGGCTCTCGCATCG

310 320 330 340 350 360

| | | | | |

*Ta*D ATGAAGAACGTAGCGAAATGCGATACTTGGTGTGAATTGCAGAATCCCGTGAACCATCGA

*Ta*K ATGAAGAACGTAGCGAAATGCGATACTTGGTGTGAATTGCAGAATCCCGTGAACCATCGA

*T. glaucescens* ATGAAGAACGTAGCGAAATGCGATACTTGGTGTGAATTGCAGAATCCCGTGAACCATCGA

*T. avicennioides* ATGAAGAACGTAGCGAAATGCGATACTTGGTGTGAATTGCAGAATCCCGTGAACCATCGA

*T. arenicola* ATGAAGAACGTAGCGAAATGCGATACTTGGTGTGAATTGCAGAATCCCGTGAACCATCGA

*T. catappa* ATGAAGAACGTAGCGAAATGCGATACTTGGTGTGAATTGCAGAATCCCGTGAACCATCGA

*T. benzoe* ATGAAGAACGTAGCGAAATGCGATACTTGGTGTGAATTGCAGAATCCCGTGAACCATCGA

*T. bellirica* ATGAAGAACGTAGCGAAATGCGATACTTGGTGTGAATTGCAGAATCCCGTGAACCATCGA

************************************************************

Prim.cons. ATGAAGAACGTAGCGAAATGCGATACTTGGTGTGAATTGCAGAATCCCGTGAACCATCGA

370 380 390 400 410 420

| | | | | |

*Ta*D GTCTTTGAACGCAAGTTGCGCCCGAAGCCTTGGCTGAGGGCACGTCTGCCTGGGTGTCAC

*Ta*K GTCTTTGAACGCAAGTTGCGCCCGAAGCCTTGGCTGAGGGCACGTCTGCCTGGGTGTCAC

*T. glaucescens* GTCTTTGAACGCAAGTTGCGCCCGAAGCCTTGGCTGAGGGCACGTCTGCCTGGGTGTCAC

*T. avicennioides* GTCTTTGAACGCAAGTTGCGCCCGAAGCCTTGGCTGAGGGCACGTCTGCCTGGGTGTCAC

*T. arenicola* GTCTTTGAACGCAAGTTGCGCCCGAAGCCTTGGCTGAGGGCACGTCTGCCTGGGTGTCAC

*T. catappa* GTCTTTGAACGCAAGTTGCGCCCGAAGCCTTGGCTGAGGGCACGTCTGCCTGGGTGTCAC

*T. benzoe* GTCTTTGAACGCAAGTTGCGCCCGAAGCCTTGGCTGAGGGCACGTCTGCCTGGGTGTCAC

*T. bellirica* GTCTTTGAACGCAAGTTGCGCCCGAAGCCTCGGCTGAGGGCACGTCTGCCTGGGTGTCAC

****************************** *****************************

Prim.cons. GTCTTTGAACGCAAGTTGCGCCCGAAGCCTTGGCTGAGGGCACGTCTGCCTGGGTGTCAC

430 440 450 460 470 480

| | | | | |

*Ta*D GCATCGCGTTGCATCCAAACCCTTCACCCTTCGGACGTTGCGGTGATGGTCTGGATGCGG

*Ta*K GCATCGCGTTGCATCCAAACCCTTCACCCTTCGGACGTTGCGGTGATGGTCTGGATGCGG

*T. glaucescens* GCATCGCGTTGCATCCAAACCCTTCACCCTTCGGACGTTGCGGTGACGGTCTGGATGCGG

*T. avicennioides* GCATCGCGTTGCATCCAAACCCTTCACCCTTCGGACGTTGCGGTGACGGTCTGGATGCGG

*T. arenicola* GCATCGCGTTGCCTCCAAACCCTTCACCCTTCGTTCGTTGCGGTGATGGTCTGGATGCGG

*T. catappa* GCATCGCGTTGCCTCCAAACCCTTCACCCTTCGTTCGTTGCGGTGATGGTCTGGATGCGG

*T. benzoe* GCATCGCGTTGCCTCCAAACCCTTCACCCTTCGAACGTTGCGGTGATGGTCTGGGTGCGG

*T. bellirica* GCATCGCGTTGCCTCCATACCCTCCACCCCTCGAGCGATGGGGAGACGGTCCGGAAGCGG

************ **** ***** ***** *** ** ** ** ** **** ** ****

Prim.cons. GCATCGCGTTGC2TCCAAACCCTTCACCCTTCGGACGTTGCGGTGATGGTCTGGATGCGG

490 500 510 520 530 540

| | | | | |

*Ta*D AAGCTGGCCTCCCGCGGCCGCGAGCCACGGATGGCCCAAACACGTGCTAGGGGAGCGAAG

*Ta*K AAGCTGGCCTCCCGCGGCCGCGAGCCACGGATGGCCCAAACACGTGCTAGGGGAGCGAAG

*T. glaucescens* AAGCTGGCCTCCCGCGGCCGCGAGCCACGGATGGCCCAAACACGTGCTAGGGGAGCGAAG

*T. avicennioides* AAGCTGGCCTCCCGCGGCCGCGAGCCACGGATGGCCCAAACACGTGCTAGGGGAGCGAAG

*T. arenicola* AAGCTGGCCTCCCGCGGCCACGAGCCACGGATGGCCCAAACACGTGCTAGGGAAGCGAAG

*T. catappa* AAGTTGGCCTCCCGCGGCCACGAGCCACGGATGGCCCAAACACGTGCTAGGGAAGCGAAG

*T. benzoe* AAGCTGGCCTCCCGCGGCCACTAGCCACGGATGGCCCAAACACGTGCTAGGGAAGCGAAG

*T. bellirica* AAGCTGGCCTCCCGTGACCACGAGCCACGGATGGCCCAAATACGCGCTGGGGAAGCAAAG

*** ********** * ** * ****************** *** *** *** *** ***

Prim.cons. AAGCTGGCCTCCCGCGGCC2CGAGCCACGGATGGCCCAAACACGTGCTAGGG2AGCGAAG

550 560 570 580 590 600

| | | | | |

*Ta*D CGCCACGGCATTCGGTGGTTGATCCAAGCCCCAGAAGCAGTGCCGGCGGTGGCCGCACCC

*Ta*K CGCCACGGCATTCGGTGGTTGATCCAAGCCCCAGAAGCAGTGCCGGCGGTGGCCGCACCC

*T. glaucescens* CGCCACGGCATTCGGTGGTTGATCCAAGCCCCAGAAGCAGTGCCGGCGGTGGCCGCACCC

*T. avicennioides* CGCCACGGCATTCGGTGGTTGATCCAAGCCCCAGAAGCAGTGCCGGCGGTGGCCGCACCC

*T. arenicola* CGCCACGGCATTCGGTGGTTGATCCAAGCCCCAGAAGCAGTGCCGGCGGTGGCCGCGTCT

*T. catappa* CGCCACGGCATTCGGTGGTTGATCCAAGCCCCAGAAGCAGTGCCGGCGGTGGCCGCGTCT

*T. benzoe* CGCCACGGCATTCGGTGGTTGATCCAAGCCCCAGAAGCAGTGCCGGTGGTGGCCGCATCT

*T. bellirica* CGCCACGGCATTCGGTGGTCGATCCGAGCCCCAGAAACAGTGCCCGTGGCGGCCGCATCC

******************* ***** ********** ******* * ** ****** *

Prim.cons. CGCCACGGCATTCGGTGGTTGATCCAAGCCCCAGAAGCAGTGCCGGCGGTGGCCGCA2CC

610 620 630 640 650 660

| | | | | |

*Ta*D GTCCCTAGCCGACGACCCTAAACGTTAACCAACGCGACCTCAGGTCAGGCGGGGCTACCC

*Ta*K GTCCCTAGCCGACGACCCTAAACGTTAACCAACGCGACCTCAGGTCAGGCGGGGCTACCC

*T. glaucescens* GTCCCTAGCCGACGACCCTAAACGTTAACCAACGCGACCTCAGGTCAGGCGGGGCTACCC

*T. avicennioides* GTCCCTAGCCGACGACCCTAAACGTTAACCAACGCGACCTCAGGTCAGGCGGGGCTACCC

*T. arenicola* GTCCTTAGCCCACGACCCTAAACGTTAACCAACGCGACCTCAGGTCAGGCGGGGCTACCC

*T. catappa* GTCCTTAGCCTACGACCCTAAACGTTAACCAACGCGACCTCAGGTCAGGCGGGGCTACCC

T*. benzoe* GTCCCTAGCCCACGACCCTAAACGTTAACCAACGCGACCTCAGGTCAGGCGGGGCTACCC

*T. bellirica* GTCCCCAGCCGACGGCCCTAAACGTTAACCAACGCGACCTCAGGTCAGGCGGGGCTACCC

**** **** *** *********************************************

Prim.cons. GTCCCTAGCCGACGACCCTAAACGTTAACCAACGCGACCTCAGGTCAGGCGGGGCTACCC

TermRev

670 680 690

| | |

*Ta*D GCTGAGTTTAAGC------------------

*Ta*K GCTGAGTTTAAGC------------------

*T. glaucescens* GCTGAGTTTAAGCATATCAATAAGCGGAGGA

*T. avicennioides* GCTGAGTTTAAGCATATCAATAAGCGGAGGA

*T. arenicola* GCTGAGTTTAAGCATATCAATAAGCGGAGGA

*T. catappa* GCTGAGTTTAAGCATATCAATAAGCGGAGGA

*T. benzoe* GCTGAGTTTAAGCATATCAATAAGCGGAGGA

*T. bellirica* GCTGAGTTTAAGCATATCAATAAGCGGAGGA

************

Prim.cons. GCTGAGTTTAAGCATATCAATAAGCGGAGGA
